# Fabrication of Metal-Substituted Polyoxometalates for Colorimetric Detection of Dopamine and Ractopamine

**DOI:** 10.3390/ma11050674

**Published:** 2018-04-26

**Authors:** Xixin Duan, Zhixian Bai, Xueting Shao, Jian Xu, Ning Yan, Junyou Shi, Xiaohong Wang

**Affiliations:** 1 Jilin Provincial Key Laboratory of Wooden Materials Science and Engineering, Beihua University, Jilin 132013, China; duanxixin@hotmail.com (X.D.); baizhixian@hotmail.com (Z.B.); chemistrylovers@163.com (X.S.); ning.yan@utoronto.ca (N.Y.); 2 Key Lab of Polyoxometalate Science of Ministry of Education, Northeast Normal University, Changchun 130024, China; z853182371@163.com; 3 Department of Chemical Engineering and Applied Chemistry, University of Toronto, Toronto, ON M5S 3B3, Canada

**Keywords:** polyoxometalates, peroxidase-like, colorimetric detection, dopamine, ractopamine

## Abstract

A novel colorimetric detection method based on the peroxidase-like activity of metal-substituted polyoxometalates (POMs) of SiW_9_M_3_ (M = Co^2+^, Fe^3+^, Cu^2+^, Mn^2+^) has been established. POMs can catalyze oxidation of dopamine (DA) and ractopamine (RAC) by H_2_O_2_ in aqueous solutions. SiW_9_Co_3_-based POMs detect DA at concentrations as low as 5.38 × 10^−6^ mol·L^−1^ simply by observation of the color change from colorless to orange using the naked eye. RAC is detected by observing the change from colorless to slight red by SiW_9_Cu_3_ with a detection limit of 7.94 × 10^−5^ mol·L^−1^. This study shows that colorimetric DA and RAC detection using SiW_9_Co_3_ and SiW_9_Cu_3_ is highly selective and sensitive as well as visually observable.

## 1. Introduction

*β*-agonists are known as phenylethanolamines with different substituent groups on the aromatic ring and the terminal amino group. Usually, *β*-agonists are applied for the treatment of pulmonary disease and asthma. In recent years, *β*-agonists have been illegally used to promote animal growth and increase the percentage of lean meat in pig carcasses. However, the excessive addition of *β*-agonists in animal feed results in accumulation in human tissue after consumption of the defective meat, which can lead to acute or chronic poisoning. Therefore, there is a need to develop fast, easy, selective, and accurate methods for detecting *β*-agonists for protecting public health.

Dopamine (DA) and ractopamine (RAC) are the two main *β*-agonists in the list of banned feed additives in China and European Union (EU). A series of approaches have been developed for detecting DA, such as electrochemical analysis [1], high performance liquid chromatography (HPLC) [2], and the chemiluminescence method [3]. Methods for the detection of RAC have also been established, including capillary electrophoresis [4], HPLC [5], enzyme-linked immunoassay (ELISA) [6], electrochemical detection [7], and colloidal gold [8]. However, these methods involve complicated sample treatment procedures and have poor reproductivity. In the past decades, colorimetric detection, as one convenient approach, has been explored for providing naked-eye readout signals with simple and rapid detection assays. However, the activity and selectivity of catalysts are of crucial importance for the colorimetric detection.

In recent years, various inorganic nanomaterials have been found to have activities much like natural enzymes, which can catalyze chemical reactions in a series of physiological conditions. The activity of nano-enzymes is similar to that of natural enzymes and they both are active under physiological conditions. However, nano-enzymes have several attractive properties: they have stable activity with convenient active adjustment, and are cheap, with easy preparation. It has been found that Au nanoparticles (AuNPs) possess intrinsic peroxidase-like activity, which can catalyze oxidation substrates with H_2_O_2_ to develop colored compounds. Recently, AuNPs have been shown to oxidize the peroxidase substrate of 3,3′,5,5′-tetramethylbenzidine (TMB) in the presence of H_2_O_2_, and colored products are generated [9]. Nowadays, the application of AuNPs as peroxidase nanomimetics has gained increasing attention. They have good stability and are easily prepared, with flexibility of design for the colorimetric detection of DA through the color change associated with aggregation (red-to-purple or blue) and redispersion (purple-to-red) [10]. However, the resulting color change cannot be easily observed using naked-eye readouts, and requires a measurement instrument. A novel, rapid, and sensitive detection system is needed in this field.

Polyoxometalates (POMs) are a distinctive class of inorganic metal–oxygen clusters, which have wide applications in catalysis, materials, and nanotechnology because their transition metals are in the highest oxidation states. Therefore, POMs can show diverse peroxidase-like activity by changing the type of metals in their structure. Due to their intrinsic oxidase activity, POMs have played important roles in the colorimetric detection of cancer cells [11,12], H_2_O_2_ [13], and various other biomolecules. For the first time, Wang et al. [14] found that H_3_PW_12_O_40_ exhibited intrinsic peroxidase-like activity and the system of POMs/H_2_O_2_/TMB could be used for the detection of H_2_O_2_ or glucose. Then, iron-substituted POMs as peroxidases were prepared for colorimetric immunoassays of H_2_O_2_ [13] and cancer cells [12]. In recent years, progress has been made in the detection of DA with POMs by electrochemical biosensors [15,16,17]. However, to the best of our knowledge, few investigations have focused on the utilization of POMs as colorimetric immunoassay agents for rapid and sensitive chromogenic detection of DA and RAC.

Inspired by this concept, the adjustable peroxidase-like activity of POMs is proposed to be superior to that of AuNPs in the colorimetric detection of DA and RAC. Here, different metal-substituted polyoxometalates are prepared for colorimetric detection of DA and RAC. The four main findings are as follows. Firstly, the peroxidase-like activity of POMs was adjustable by varying POM structures and the substitution of transition metals, which led to changing the oxidation state of different series of POMs. Secondly, the types of metals in the structure of POMs were very important for the selective oxidation of DA and RAC. The number of metals contributed to the sensitivity of the detection system. Thirdly, the utilization of POMs could overcome the issue of the color change not being easily observable when AuNPs were used for the chromogenic detection of DA. Few studies in the literature have focused on the colorimetric detection of RAC and DA by POMs with H_2_O_2_. Fourthly, a novel method was established in this study for the colorimetric detection of RAC and DA, which was simple, rapid, easy to observe, and highly selective and sensitive.

## 2. Materials and Methods 

### 2.1. Preparation of Catalysts

POMs of K_4_SiW_12_O_40_ (SiW_12_), K_8_SiW_11_O_39_·13H_2_O (SiW_11_), K_8_[γ-SiW_10_O_36_]·12H_2_O (SiW_10_), and *α*-Na_10_SiW_9_O_34_·18H_2_O (SiW_9_) were synthesized according to the method previously reported [18]. Catalysts of SiW_11_M, SiW_10_M_2_ and SiW_9_M_3_ (M = Co^2+^, Fe^3+^, Cu^2+^, Mn^2+^) were prepared by methods described in [19,20,21], respectively. The detailed procedure of catalysts was described in the supporting information. Chemical reagents were all of analytical grade and used without further purification. Aqueous solutions were prepared from deionized water. DA (98%), glycine, alanine, glucose, and urea were purchased from Aladdin (Shanghai, China). RAC (97%) was provided by J&K Scientific LTD (Hong Kong, China).

### 2.2. Procedure for the Detection of RAC

POMs (8 mM) and H_2_O_2_ (200 mM) were added into an aqueous solution of 1 mL. RAC (5 mM) was added into the above mixture, and then blended again. Reaction time was from 0 to 25 min. The sample solution was measured at 515 nm by a Shimadzu (Kyoto, Japan) UV-2700 UV-VIS spectrophotometer (200–600 nm). 

### 2.3. Procedure for the Detection of DA

POMs (10 mM) and H_2_O_2_ (100 mM) were added into an aqueous solution of 1 mL. DA (8 mM) was added into the above mixture, and then blended again. The mixed solution was laid aside for 0–15 min. The resulting solution was measured at 475 nm by a Shimadzu UV-2700 UV-VIS spectrophotometer (200–600 nm). 

### 2.4. Other Analytical Measurements

FT-IR spectra (4000–400 cm^−1^) were collected using KBr discs on a Nicolet Magna (Madison, America) 560 IR spectrometer. XRD (X-ray diffraction) patterns of catalysts were characterized by Japan Rigaku Dmax (ToKyo, Japan) 2000 X-ray diffractometer with Cu Kα radiation (λ = 0.154178 nm). XPS (X-ray photoelectron spectroscopy) spectra were recorded on an Escalab-MK II (London, England) photoelectronic spectrometer with Al Kα (1200 eV). 

## 3. Results

### 3.1. Characterization of Catalysts

FT-IR and UV-VIS were used to identify the different structural among SiW_11_M, SiW_10_M_2_, and SiW_9_M_3_ in Table S1. These three kinds of POMs are all derivatives of Keggin POMs. The typical peaks of UV-VIS at 200 nm and 250 nm were attributed to the transition of O_d_-W and O_b_/O_c_→W, respectively, in the heteropolyacid anion. This was in accordance with the characteristic peaks of the Keggin heteropolyanions.

FT-IR results indicated that peaks in the range of 600–1100 cm^−1^ corresponded to the asymmetric vibrations of P-O_a_, W-O_d_, W-O_b_, and W-O_c_ of the Keggin structure [22]. This confirmed the compounds all maintained the Keggin structure. However, the FT-IR spectrum of SiW_9_M_3_ (Figure S1) differed from those of SiW_11_M and SiW_10_M_2_ due to the splitting of the peak at 600–800 cm^−1^ [21], and the particular results from the vibration of W-O_c_-W corresponded to the structure of SiW_9_M_3_. 

XRD patterns were collected to characterize the solid structure of SiW_9_M_3_, SiW_9_, SiW_10_, SiW_11_, and SiW_12_ in Figure S2. The obvious peaks of SiW_9_, SiW_10_, SiW_11_, and SiW_12_ could be observed due to their distinctive crystal structures. As derivatives of Keggin structure, catalysts of SiW_9_M_3_ also kept similar diffraction peaks to pure SiW_12_ (18.14°, 20.56°, 25.34°, 29.32° and 34.78°) [23]. This indicated that the primary structure of heteropolyacids remained in the series of polyoxometalates. The difference was attributed to the introduction of different metals ions.

The binding energy of SiW_9_M_3_ in the spectra of XPS gave the peaks of W 4f, Si 2p, K 2p and O 1s at 35.08, 102.8, 284.08, and 531.089 eV, respectively, confirming that elements of O and W existed as O^2−^ and W^6+^ (Figure S3a) [24,25] High-resolution spectra of different metal ions are shown in Figure S3b–e, which corresponded to the energy of Co^2+^, Cu^2+^, Fe^3+^, and Mn^2+^ in the catalysts of SiW_9_M_3_.

### 3.2. Catalytic Activity of POM Catalysts

POMs are distinctive due to their fast and reversible multi-electron redox processes, even under wild conditions. The redox property of POMs was adjusted by the type and amount of metal ions to complete the reaction with different substrates. In our investigation, it has been confirmed that the type and number of transition metal ions had a crucial effect on the catalytic results when the different POMs catalysts were used for the detection of DA and RAC.

**Figure 1:**
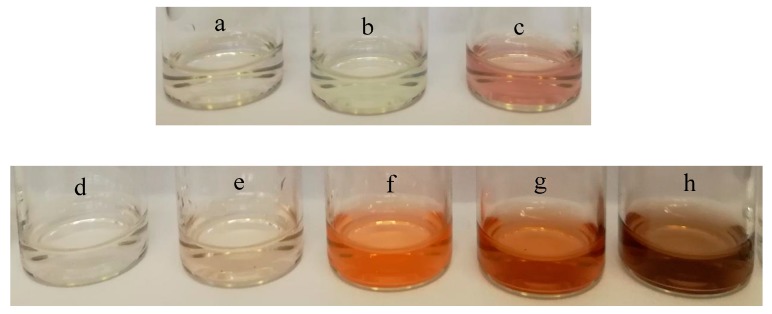
Photographs of detection of ractopamine (RAC; top) by SiW_9_Cu_3_ and dopamine (DA; bottom) by SiW_9_Co_3_; (**a**) RAC + H_2_O_2_ + 25 min; (**b**) RAC + SiW_9_Cu_3_ + 25 min; (**c**) RAC + SiW_9_Cu_3_ + H_2_O_2_ + 25 min; (**d**) DA + H_2_O_2_ + 10 min; (**e**) DA + SiW_9_Co_3_ + 10 min; (**f**) DA + SiW_9_Co_3_ + H_2_O_2_ + 10 min; (**g**) DA + SiW_9_Co_3_ + H_2_O_2_ + 15 min; (**h**) DA + SiW_9_Co_3_ + H_2_O_2_ + 30 min.

**Figure 2:**
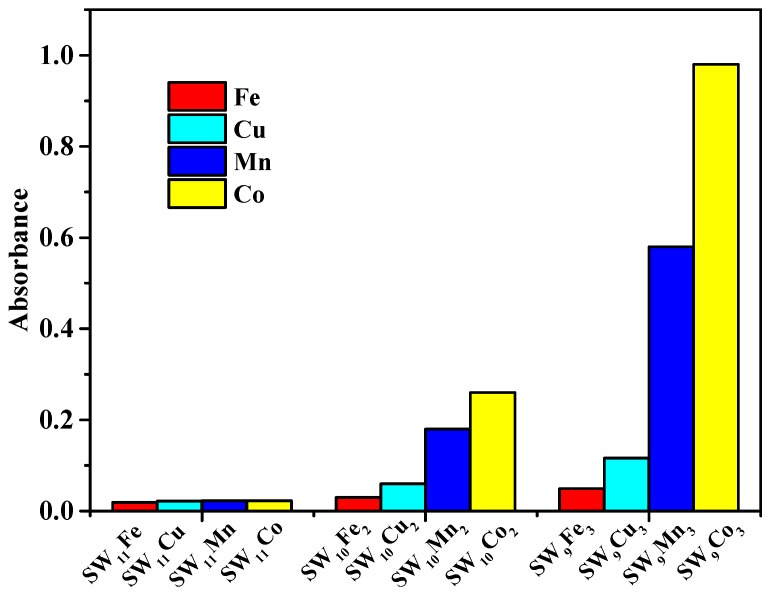
Comparison of SiW_11_M, SiW_10_M_2_ and SiW_9_M_3_ catalysts for the detection of DA at 475 nm in aqueous solutions with 60 µL H_2_O_2_, 120 µL DA, and 80 µL of catalyst, with a reaction time of 10 min.

For the detection of RAC, only the tri-substituted catalyst of SiW_9_Cu_3_ affected the colorimetric detection and provided the obvious color change from colorless to slight red (Figure 1 top). Hence, the subsequent detections of RAC were all carried out by SiW_9_Cu_3_. For the detection of DA, the catalytic activity (Figure 2) was in the order SiW_9_M_3_ > SiW_10_M_2_ > SiW_11_M. This indicated that the increasing number of transition metal ions led to the stronger redox property of POMs, which was beneficial to the colorimetric detection of DA. The effect of metal ions was in the order of Co > Mn > Cu > Fe, and SiW_9_Co_3_ exhibited the highest catalytic performance from colorless to orange (Figure 1 bottom). Hence, the following detection studies of DA were all carried out by SiW_9_Co_3_. The above results confirmed that the type of transition metal ions had an effect on the catalytic performance and selectivity when different POM catalysts were used for the detection of DA. The obvious color change was easy for visual discrimination, and showed a superior performance to that of AuNPs.

### 3.3. Detection of RAC 

**Figure 3:**
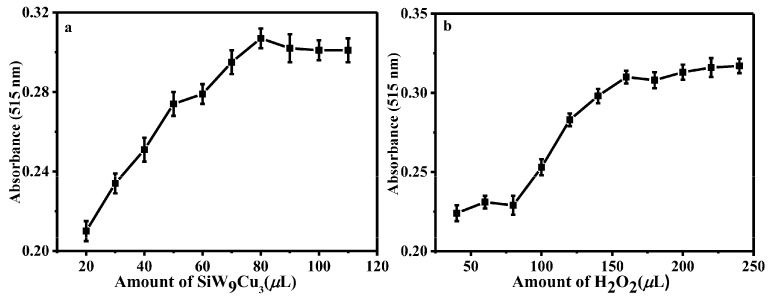
Effect of amount of SiW_9_Cu_3_ (**a**) and H_2_O_2_ (**b**) in the detection of RAC. (**a**) 100 µL RAC, 160 µL H_2_O_2_; and (**b**) 80 µL SiW_9_Cu_3_, 100 µL RAC, and reaction time of 25 min.

In this study, the effect of the amount of SiW_9_Cu_3_ (Figure 3a) was in the range of 20–110 µL. It was investigated at room temperature. The absorbance increased with the concentration of the catalyst, which illustrated that the POM detection system was highly specific for RAC. The performance at the concentration of 4.78 × 10^−4^ mol·L^−1^ (80 µL) was the highest, and the absorbance of 90–110 µL was slightly decreased. Hence, 80 µL of catalyst was chosen for the naked-eye observation of RAC.

As a key factor, the oxidant content of H_2_O_2_ has decisive influences on the catalysis of RAC. Results are shown in Figure 3b and increasing amounts of H_2_O_2_ caused increasing absorption. When the concentration reached 2.39 × 10^−2^ mol·L^−1^ (160 µL), the maximum absorption was obtained. However, with further increasing the amount of H_2_O_2_, there was no obvious improvement in absorbance.

**Figure 4:**
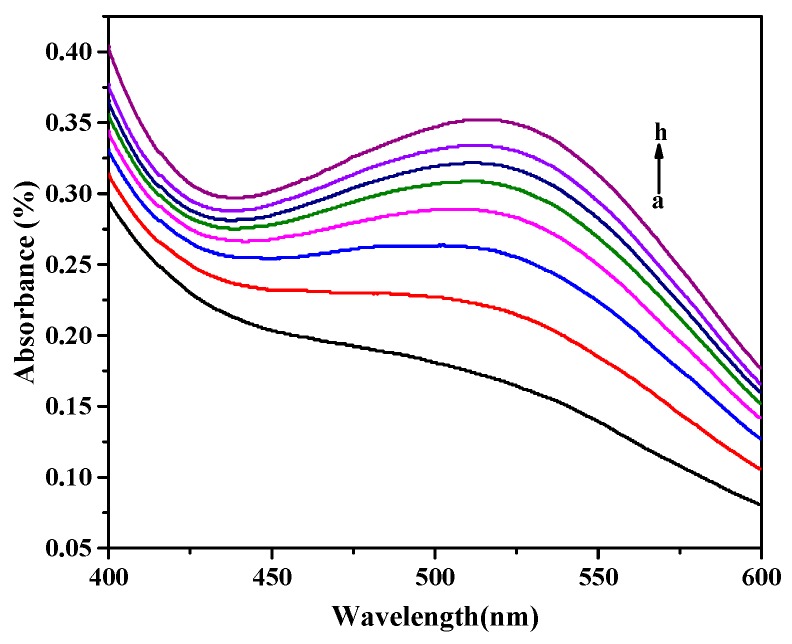
UV−VIS spectra of RAC catalyzed by SiW_9_Cu_3,_ with 80 µL SiW_9_Cu_3_, 100 µL RAC, and 160 µL H_2_O_2_. Reaction times (a) 5, (b) 10, (c) 15, (d) 20, (e) 25, (f) 30, (g) 35, and (h) 60 min.

The absorption of samples was tested at room temperature every 5 min and the longest reaction time was 60 min (Figure 4). The obvious color change was observed from colorless to slight red at just under 5 min. After prolonging the response time, an increasing absorbance was obtained, but an apparent deepening of color was not observed. When reaction time was 25 min, the shape of absorption curve was significantly superior to the others. At an even longer time, the absorbance showed a slow increase and the curve shape kept constant. Thus, the optimum reaction time was determined as 25 min. The color of the solution was also stable even if the solution was laid aside for 60 min, which indicated that this system was suitable for the colorimetric detection of RAC with SiW_9_Cu_3_ in aqueous solution.

**Figure 5:**
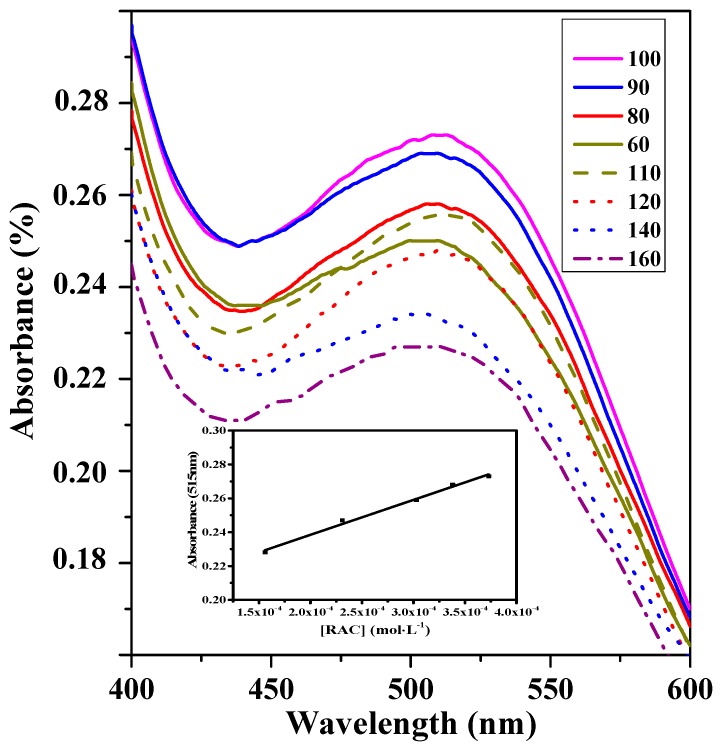
UV−VIS spectra of the amount of RAC with 80 µL SiW_9_Cu_3_, 160 µL H_2_O_2_, and reaction time of 25 min. Insert: the plot of absorbance at 515 nm versus the concentration of RAC.

Figure 5 illustrated the amount of RAC had an effect on the colorimetric change. In the series of experiments, the amount of RAC was varied in the range of 60 to 160 µL and the solutions all showed a slight red color, which proved that the new system was suitable for qualitative detection of RAC with obvious color change. The maximum absorption was obtained at 100 µL of RAC. On further increasing the usage of RAC, the decreasing absorbance could be observed, which was attributed to the decreasing concentration of active catalyst sites and the shortage of oxidizing capacity of the system with increasing amounts of RAC. The linear range was in the range from 1.56 × 10^−4^ to 3.73 × 10^−4^ mol·L^−1^ and the limit of detection was 7.94 × 10^−5^ mol·L^−1^. Therefore, the optimum conditions could be considered as 80 µL SiW_9_Cu_3_, 100 µL RAC, and 160 µL H_2_O_2_, with a reaction time of 25 min.

### 3.4. DA Detection 

As *β*-agonists and the most important catecholamine neurotransmitters, DA has crucial effects in the human central nervous system under physiological conditions. Hence, the following experiments were all conducted in aqueous solutions, representing a wide range of application conditions in the biometric technology, medical, and pharmaceutical fields. 

**Figure 6:**
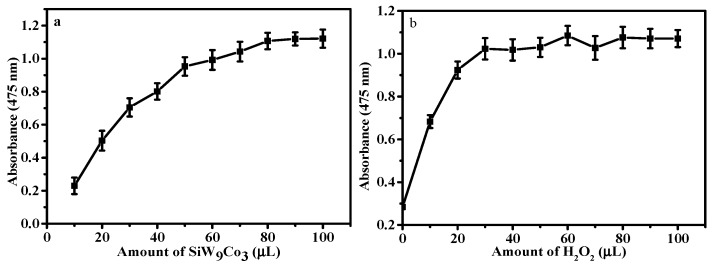
Effect of the amount of SiW_9_Co_3_ (**a**) and H_2_O_2_ (**b**) for the detection of DA. (**a**) 120 µL DA, 60 µL H_2_O_2_; and (**b**) 80 µL SiW_9_Co_3_, 120 µL DA, and reaction time of 10 min.

Figure 6a illustrates the effect of the concentration of SiW_9_Co_3_ on the oxidation of DA at room temperature. When the usage of SiW_9_Co_3_ was in the range of 0–7.81 × 10^−4^ mol·L^−1^, the absorbance increased with the concentration of catalyst, which illustrated that the system was highly specific to DA. The concentration of 6.35 × 10^−4^ mol·L^−1^ (80 µL) was considered as the optimum usage amount.

The influence of H_2_O_2_ (Figure 6b) was also detected at room temperature in the experiment. The absorption increased remarkably with the concentration of oxidant. The maximum absorption was observed at 4.76 × 10^−3^ mol·L^−1^ (60 µL). Then, the results were slightly decreased by increasing the concentration of H_2_O_2_. This indicated that the amount of H_2_O_2_ was crucial in the experiment.

**Figure 7:**
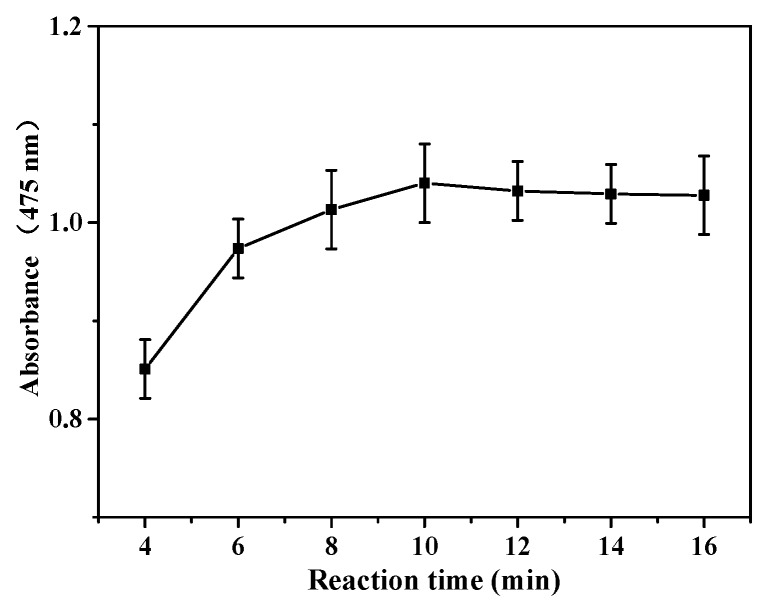
Effect of reaction time with 80 µL SiW_9_Co_3_, 120 µL DA, and 60 µL H_2_O_2_.

The appropriate reaction time is also one of the necessary reaction conditions for the colorimetric detection of DA. The results of absorbance (Figure 7) increased with the prolonged reaction time and the optimum time was 10 min. However, with further increasing reaction time, the color of solution was changed from orange to dark brown, which was less observable using the naked eye for readouts. 

It was confirmed that SiW_9_Co^II^_3_ was oxidized by H_2_O_2_ and then the product of SiW_9_Co^III^_m_Co^II^_3-m_ reacted with DA by the reaction of catalytic oxidation [26,27]. As a result, the orange aminochrome (AC) was formed, which was detected at 475 nm with UV-VIS spectrometer, and could be applied for colorimetric detection. Figure 1 (bottom photographs) demonstrates the colorimetric process for DA with SiW_9_Co_3_ and an obvious color change could be observed from colorless to orange by the naked eye within an appropriate reaction time. However, a longer reaction time was not beneficial to the color observation because the AC can regroup as 5,6-dihydroxyindole (DHI) which was further oxidized to indole-5,6-quinone (IQ). Finally dark brown neuromelanin was generated. 

**Figure 8:**
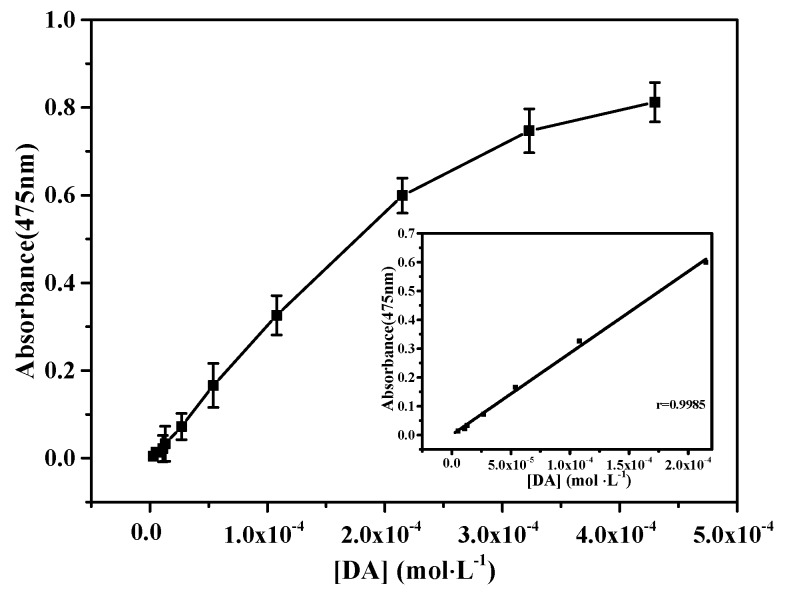
The linear range for colorimetric detection of DA with SiW_9_Co_3_. Insert: the plot of absorbance at 475 nm versus the concentration of DA. Experiments: 80 µL SiW_9_Co_3_, 60 µL H_2_O_2_, and reaction time of 10 min.

The concentration of DA could be detected by SiW_9_Co_3_ under the optimum reaction condition. As shown in Figure 8, the calibration plot for absorbance at 475 nm against catalyst activity of DA was detected with a linear range from 1.08 × 10^−4^ to 5.38 × 10^−6^ mol·L^−1^. The linear relationship indicated that the detection was kinetically controlled by DA and thus the reporting system could be used for the DA activity assay.

According to CODEX, the highest limitation of RAC is 2.96 × 10^−8^ mol·L^−1^ (10 ppb). DA is also one of the main *β*-agonists. Thus, studies [28,29,30,31,32,33,34] have been put forward (Table 1). In recent years, electrochemical biosensors [15,16,17] based on POMs have made a rapid progress in the detection of DA. Compared with the other results in the colorimetric detection of DA and RAC, SiW_9_Co_3_ is shown to represent a rapid simple detective system for DA with the addition of only H_2_O_2_. In recent years, few studies have reported on the use of POMs for the colorimetric detection of RAC. A new detection system of RAC was put forward in this study that was simple, easily observable using the naked eye, and stable over time. 

### 3.5. Interference Detection

**Figure 9:**
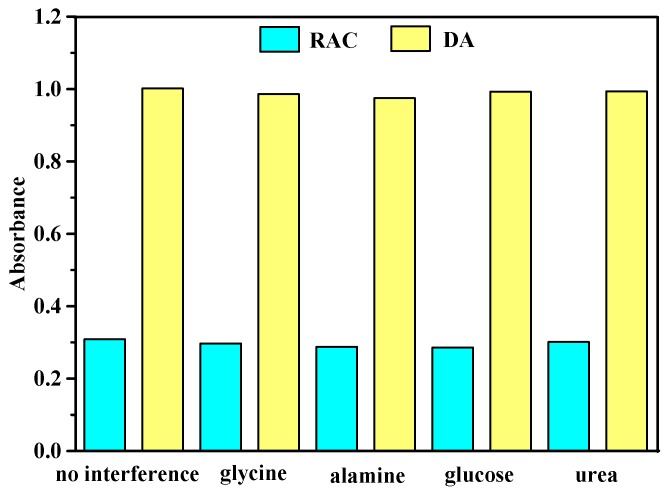
Comparison of the effect of interferences on the colorimetric detection of RAC and DA. Experiments for RAC: 100 µL interference (5 mM), 80 µL SiW_9_Cu_3_, 100 µL RAC, 160 µL H_2_O_2_, and reaction time of 25 min. Experiments for DA: 120 µL interference (8 mM), 60µL H_2_O_2_, 120µL DA, 80µL SiW_9_Co_3_, and reaction time of 10 min.

The amino group was effective in the structure of DA and RAC for colorimetric detection [35]. In order to validate the reliability of the proposed method, the influence of the other molecules containing the amino group should be discussed in the sample detection. Four chemicals, including glycine, alanine, glucose, and urea, were selected as interfering substances to evaluate the selectivity of catalysts, and results are presented in Figure 9. It was evident that interfering substances did not lead to a higher absorbance, which revealed that the system was highly selective for DA and RAC. Therefore, it was clear that SiW_9_Co_3_ and SiW_9_Cu_3_ were specific for the colorimetric detection of DA and RAC, respectively.

## 4. Conclusions

Novel detection systems for DA and RAC have been established with POMs with highly visible color change for the first time. SiW_9_Co_3_ exhibited a color change from colorless to orange with H_2_O_2_ for the detection of DA. SiW_9_Cu_3_ produced a color change from colorless to slight red for the detection of RAC with H_2_O_2_. SiW_9_Co_3_ and SiW_9_Cu_3_ both led to a more convenient method for observation using the naked eye only. Therefore, these catalysts have great application potential for the detection of DA and RAC in aqueous solutions, and the corresponding detection system is simple, rapid, selective, and sensitive to DA and RAC. 

## Figures and Tables

**Table 1:** Comparison of DA and RAC obtained by different colorimetric catalysts in recent years. AuNPs: Au nanoparticles. AgNRs: Ag nanoparticles TMB: 3,3′,5,5′-tetramethylbenzidine. DBA: Dopamine-binding aptamer. UF: Un-functionalized. MA: Melamine. TGA: Thioglycolic acid.

Catalyst	Detection	Time (min)	Linear Range (mol/L)	Detection Limit (mol/L)	Reference
CoFe_2_O_4_/CoS	DA (TMB + H_2_O_2_)	10	0–5 × 10^−5^	5.8 × 10^−7^	[28]
AuNPs	DA (NaOH + TGA)	20	0–10^−6^	5.7 × 10^−7^	[29]
AuNPs	DA (DBA + NaCl)	20	5.4 × 10^−7^–5.4 × 10^−6^	3.6 × 10^−7^	[30]
Au-AgNRs	DA	25 (70 °C)	0.20–12	0.047	[31]
BSA-AuNCs	DA (TMB + H_2_O_2_)	10	1 × 10^−8^–1 × 10^−3^	1 × 10^−8^	[32]
UF-AuNPs	DA	5	5 × 10^−7^–5 × 10^−4^	5 × 10^−7^	[33]
SiW_9_Co_3_	DA (H_2_O_2_)	10	1.08 × 10^−4^–5.38 × 10^−6^	5.38 × 10^−6^	This work
MA–AuNPs	RAC	10	1 × 10^−10^–5 × 10^−7^	1 × 10^−11^	[34]
SiW_9_Cu_3_	RAC (H_2_O_2_)	25	1.56 × 10^−4^–3.73 × 10^−4^	7.94 × 10^−5^	This work

## Supplementary Materials

The following are available online at http://www.mdpi.com/1996-1944/11/5/674/s1. Table S1: Results of FT-IR and UV-VIS of SiW_11_M, SiW_10_M_2_, and SiW_9_M_3_ (M = Co^2+^, Fe^3+^, Cu^2+^, Mn^2+^). Figure S1: FT-IR spectra of SiW_9_M_3_ (M = Co^2+^, Fe^3+^, Cu^2+^, Mn^2+^). Figure S2: XRD patterns of SiW_9_M_3_ (M = Co^2+^, Fe^3+^, Cu^2+^, Mn^2+^), SiW_9_, SiW_10_, SiW_11_, and SiW_12_. Figure S3: XPS spectra of SiW_9_M_3_ (M = Co^2+^, Fe^3+^, Cu^2+^, Mn^2+^). Survey (a) high-resolution of Co 2p (b), Cu 2p (c), Fe 2p (d), and Mn 2p (e). Preparation of catalysts including K_4_SiW_12_O_40_ (SiW_12_), K_8_SiW_11_O_39_·13H_2_O (SiW_11_), K_8_[γ-SiW_10_O_36_]·12H_2_O (SiW_10_), *α*-Na_10_SiW_9_O_34_·18H_2_O (SiW_9_), SiW_11_M, SiW_10_M_2_, and SiW_9_M_3_ (M = Co^2+^, Fe^3+^, Cu^2+^, Mn^2+^).
