# Supplementary material for: Fabrication of Metal-Substituted Polyoxometalates for Colorimetric Detection of Dopamine and Ractopamine
Source: Materials (Basel). 2018 Apr 26;11(5):674. doi: 10.3390/ma11050674 (PMC5978051; doi:10.3390/ma11050674)
Supplement: Supplementary file 1 [file materials-11-00674-s001.pdf]

Supplementary Materials: Fabrication of Metal-Substituted Polyoxometalates for Colorimetric Detection of Dopamine and Ractopamine

Xixin Duan [1], Zhixian Bai [1], Xueting Shao [1], Jian Xu [2], Ning Yan [1,3], Junyou Shi [1,*] and Xiaohong Wang [2,*]

Table S1. Results of FT-IR and UV-Vis of $SiW_{11}M$, $SiW_{10}M_2$, and SiW_9M_3($M = Co^{2+}$, Fe^{3+}, Cu^{2+}, Mn^{2+}).

Compound	ν/ cm^{-1}				λ/ nm	
	W-O$_d$	Si-O$_a$	W-O$_b$-W	W-O$_c$-W	O$_d$→W	O$_b$ /O$_c$→W
$SiW_{11}Fe$	1012	971	902	750	205	259
$SiW_{11}Cu$	1008	950	886	736	206	261
$SiW_{11}Mn$	999	951	885	765	203	262
$SiW_{11}Co$	1001	956	887	736	202	256
$SiW_{10}Fe_2$	973	943	841	732	203	254
$SiW_{10}Cu_2$	999	954	890	772	204	257
$SiW_{10}Mn_2$	995	950	879	789	203	260
$SiW_{10}Co_2$	995	950	887	782	201	256
SiW_9Fe_3	989	947	889	770,716,654	203	252
SiW_9Cu_3	999	939	885	770,728,639	202	253
SiW_9Mn_3	991	941	877	766,704	201	253
SiW_9Co_3	987	948	889	786,705,651	201	248

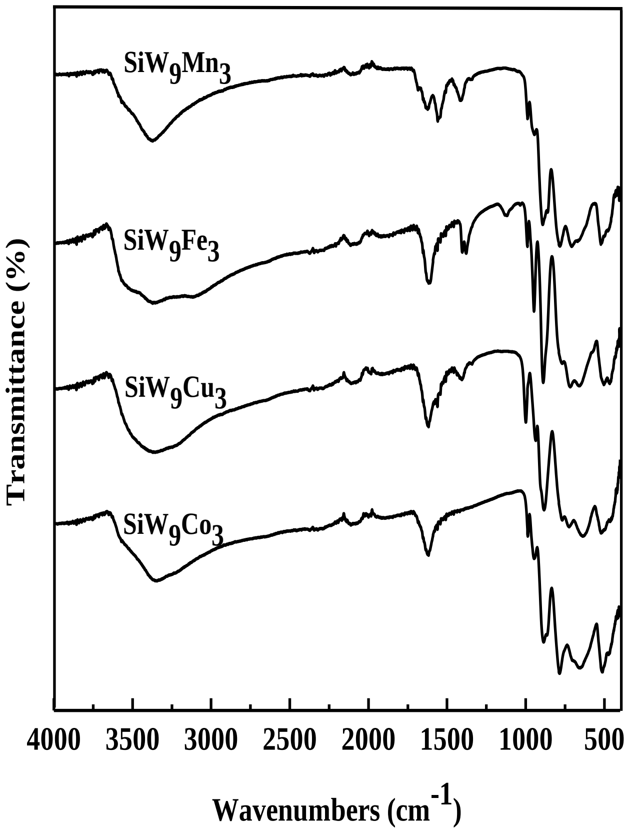

Figure S1. FTIR spectra of SiW9M3 (M = Co2+, Fe3+, Cu2+, Mn2+).

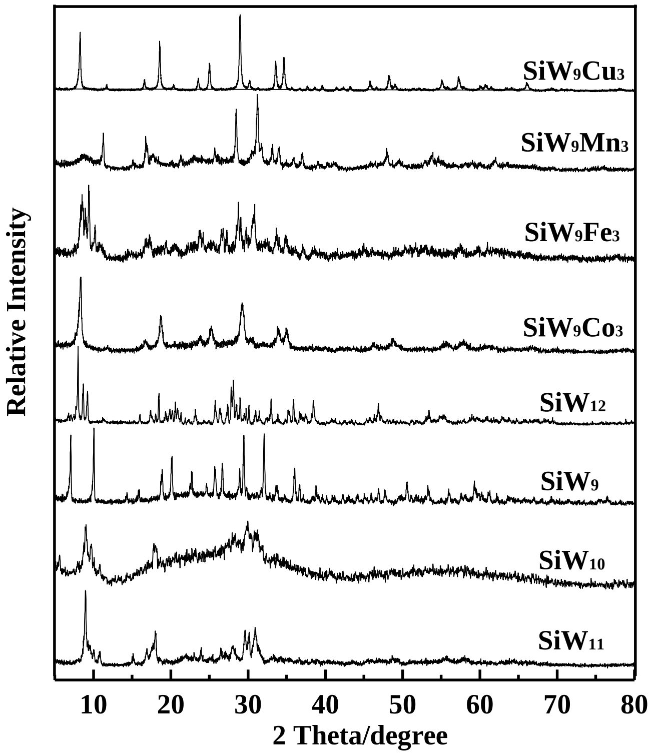

Figure S2. XRD patterns of SiW9M3 (M = Co2+, Fe3+, Cu2+, Mn2+), SiW9, SiW10, SiW11, and SiW12.

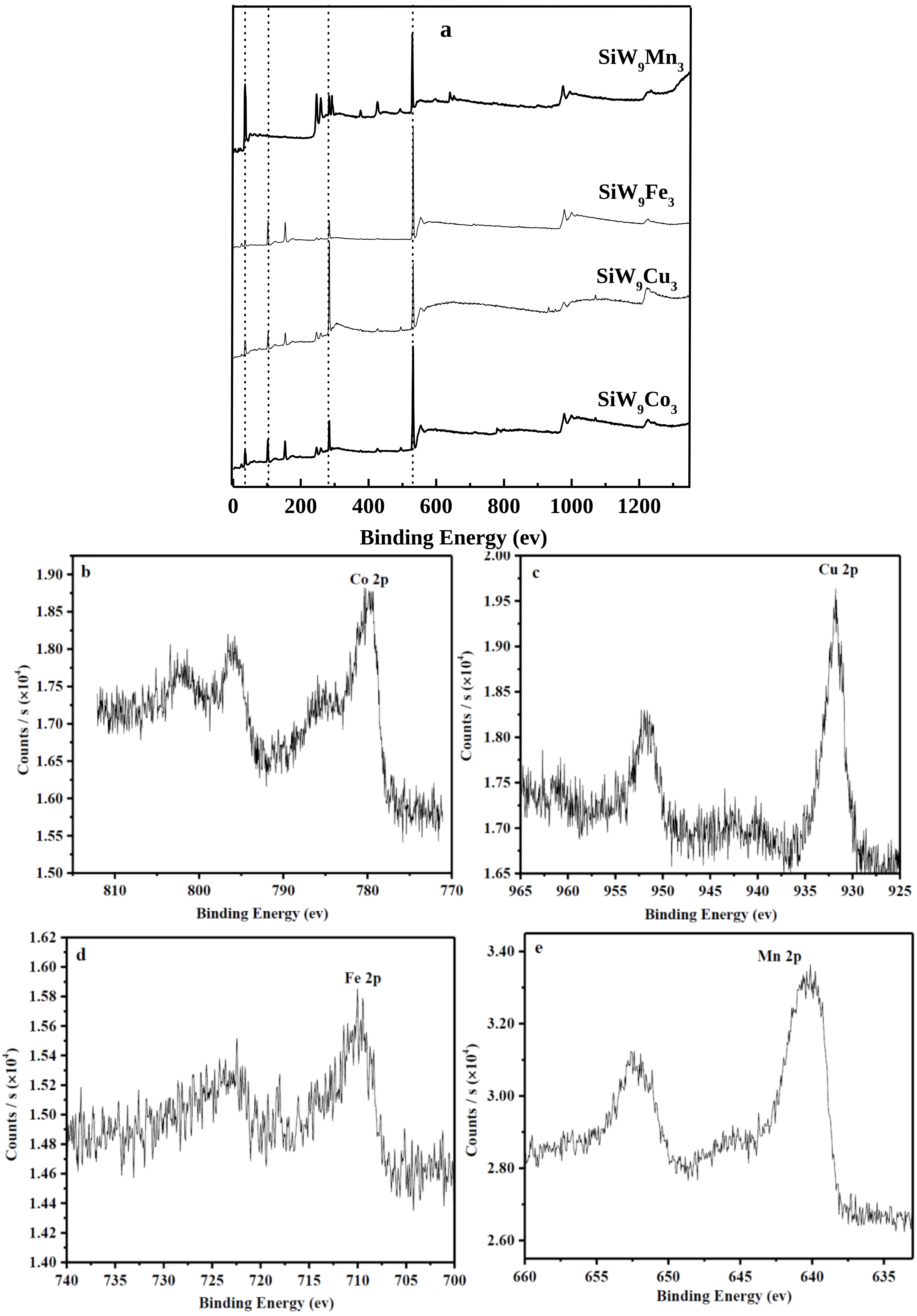

Figure S3. XPS spectra of SiW_9M_3 (M = Co^{2+}, Fe^{3+}, Cu^{2+}, Mn^{2+}). Survey (a) high-resolution of Co 2p (b), Cu 2p (c), Fe 2p (d), and Mn 2p (e).
